# The impact of leadership style in team-based primary care – staff satisfaction and motivation

**DOI:** 10.3399/BJGPO.2023.0246

**Published:** 2024-08-07

**Authors:** Sara Bhatti, Stephanie Bale, Sehar Gul, Laura Muldoon, Jennifer Rayner

**Affiliations:** 1 Alliance for Healthier Communities, Toronto, Canada; 2 Public Health Sudbury & Districts Sudbury, Ontario, Canada; 3 MPH Candidate, MGill University, Montreal, Canada; 4 Family Physician, Somerset West CHC, Ottawa, Canada; 5 Department of Family Medicine, University of Ottawa, Ottawa, Canada; 6 Centre for Studies in Family Medicine, University of Western, London, Canada

**Keywords:** team-based primary health care, leadership, job satisfaction, motivation

## Abstract

**Background:**

Leadership styles, beliefs, and behaviours are an important and critical component to the delivery of quality care in any primary care organisation. The human resource crisis in health care has resulted in greater investments in team-based care; however, some leaders may not have experience working in team-based settings.

**Aim:**

To explore what leadership characteristics, styles, and behaviours were most conducive to employee satisfaction, motivation, and delivery of care in a team-based primary care setting.

**Design & setting:**

A qualitative study involving 16 community health centre (CHC) staff from six CHCs across Ontario, Canada.

**Method:**

Thematic analysis of qualitative interviews using a framework based on transformational leadership (TL) theory.

**Results:**

The following three themes emerged from our findings as having a noticeable impact on staff motivation, morale, delivery of care, and client outcomes: transparent and open communication; opportunities to collaborate in decision making; and staff recognition and appreciation. The results of our study indicate it is critical that leaders adopt leadership styles and approaches in which every team member is informed, heard, and appreciated.

**Conclusion:**

This study described the leadership styles and characteristics that lead to improved employee satisfaction, motivation, and morale in a team-based primary care setting, and the impact this could and does have on quality and delivery of care. Future research is needed to better understand the impact of leadership in a variety of roles within a team-based environment, specifically in a multidisciplinary setting.

## How this fits in

Ontario Community Health Centres (CHCs) are community-governed, salary-based, multi-sectoral, not-for-profit organisations that have provided team-based primary health care for many decades. With the primary care crisis and need for more team-based primary care providers, leadership is critical for employee retention, satisfaction, and the delivery of quality client-centred care. However, studies exploring the impact of leadership style in team-based care settings are minimal. The findings of this study describe the potential impact and importance of the transformational leadership (TL) style, and will therefore be useful for team-based care leadership development and improvement.

## Introduction

Leadership styles have a significant influence on the functioning of healthcare systems, including creating conducive working environments, delivery of care, and patient safety to name a few.^1–3^ There are a number of different leadership styles that exist, however, within health care, and the following three styles are the most commonly referenced in the literature: transformational leadership (TL) in which leaders are clear and consistent communicators, they value the work of their staff and followers, and have the ability to inspire and motivate through appreciation and recognition of the work done by their staff; transactional leadership where leaders are focused on operational tasks and the completion of these tasks; and laissez-faire or passive leadership, which is characterised by a leader who only becomes involved where necessary, that is, management by exception.^3^

**Figure 1.**
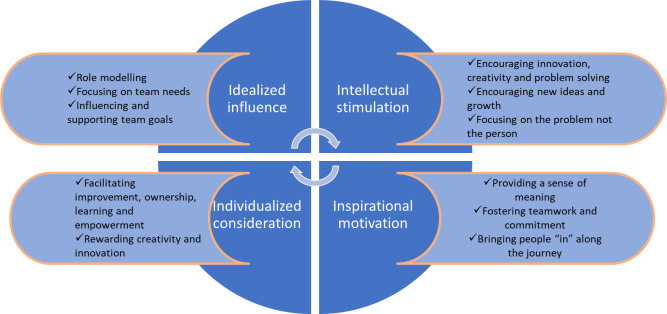
Transformational leadership theory, adapted from Educational Business Articles^4^

Existing literature describes the theory of TL as the most favourable of leadership styles.^2,3^ The theory originated in the 1970s and has continued to be adapted. It is centred around four primary elements (see Figure 1), which are as follows: idealised influence, where a leader with charisma delivers a message with tact, offers constructive feedback, and praises their staff; inspirational motivation, where a leader is motivated, speaks clearly and concisely, is upbeat and positive, and brings energy to the team; intellectual stimulation, where a leader encourages innovation, takes risks and is open to new ideas; and finally, individual consideration, where a leader recognises and values motivations, desires, and needs of its members, and learns how best to engage with it members.^4^ However, there is little literature about application of this theory in team-based primary care organisations.

Primary health care across Canada and the globe is under tremendous pressure as providers are increasingly leaving the primary care workforce. Owing to increased workloads and administrative demands, and stressors related to the COVID-19 pandemic, many providers are experiencing high levels of burnout, and are unsatisfied and unmotivated by the quality of care they are able to provide to their clients.^5,6^ This health human resource crisis has led to calls for primary care providers to work in team-based settings and governments are increasingly investing in primary care teams.^6–8^ Many of the members of these new proposed teams will be unaccustomed to working in a team-based environment, which will increase the need for information and guidance about how teams can be best led and function.

Ontario Community Health Centres (CHCs) are community-governed, salary-based, multi-sectoral not-for-profit organisations that have provided team-based primary health care for many decades. Within CHCs, primary care physicians, nurse practitioners, and nurses work alongside interprofessional team members, such as dieticians, physiotherapists, and social workers, to address patients’ health and social needs. The aim of this study was to explore how leadership characteristics were related to staff satisfaction, motivation, and delivery of care within a well-established team-based model of primary health care.

## Method

### Study design

Our previous published work explored the perceptions of different staff groups regarding team functioning (that is, team climate, organisational justice, and organisational citizenship behaviour) in Ontario CHCs.^9^ We found that some team members did not feel they were treated fairly, potentially owing to certain leadership practices. We hoped to explore this finding further by examining the causes of the perceived unfairness, including leadership practices. This study employed a grounded theory approach using semi-structured telephone interviews.

### Setting and participants

This study involved six CHCs across Ontario, Canada. CHCs serve a large number of clients with widely differing geographic and demographic characteristics, and have delivered primary health care using an interprofessional team model for >45 years. Primary care teams within CHCs report to a clinical director who then reports to the executive director of the CHC. We purposively selected the CHCs in order to maximise variation in size, rurality, and priority populations served or geographic catchment. We visited the CHCs to recruit staff members who were family physicians, nurse practitioners, nurses, interprofessional team members, and medical secretaries to participate in the study. Author JR contacted those volunteering to introduce the study and obtain consent.

### Researcher characteristics

Research team members were of different cultural and disciplinary backgrounds. LM, a CHC family physician, and JR, the research director for Ontario’s CHCs, designed the study. Interviews were conducted by a research assistant employed by the Alliance for Healthier Communities, a group representing CHCs in the province of Ontario. Coding was done by SB and SB, also employees of the Alliance for Healthier Communities.

### Data collection

We developed a semi-structured interview guide using themes identified from the literature related to the research question (for example, decision-making processes, feedback mechanisms, staff recognition and appreciation, and so on). We pilot tested the guide with CHC staff who did not work at the selected sites, ensured that the questions were clear and had face validity, and iteratively revised it to explore emerging insights from completed interviews (see Supplementary Information, Appendix 1 for interview guide). The research assistant conducted the interviews between the autumn of 2018 and winter of 2019. Interviews lasted from 30–45 minutes, and were recorded and transcribed verbatim. We conducted interviews until theme saturation was achieved, as demonstrated by limited additional elements mentioned, or once six staff members of a CHC had been interviewed.

### Analysis

Qualitative analysis was conducted in two stages. In the first stage, we used thematic analysis using an inductive approach to facilitate an understanding of the outcomes of leadership styles as experienced by staff. Then we conducted a rapid review to identify a leadership theory or style that would further guide analysis on how leadership styles might facilitate the outcomes identified within the transcripts. The TL theory was chosen as the most appropriate to understand this relationship.

During the second stage of analysis, we used Braun and Clarke’s approach to thematic analysis.^10^ This involved reading through transcripts and generating a list of initial codes using both an inductive and deductive approach (for example, themes related to TL, job satisfaction and motivation, and so on). Using the initial set of codes, one of the researchers (SB) and a research student (SB) used line-by-line coding to independently code transcripts, and compared transcripts. We then coded transcripts a second time to ensure credibility of analysis. Coders were in agreement for the majority of coding and disagreements regarding interpretation of codes were resolved between the coders. We then collated codes into themes related to TL (see Supplementary Information, Appendix 2 for coding tree). All analysis was conducted using NVivo (version 12) software.

## Results

We conducted 16 interviews within six Ontario CHCs, representing a mix of both rural and urban centres across the province (with the exception of remote sections of Northern Ontario), as well as small and large centres (based on staffing). Detailed participant information has not been provided in order to preserve their anonymity owing to the small sample size; however, participants included a mix of part- and full-time staff including three primary care providers, six nurse practitioners, five interprofessional team members, and two medical secretaries. Participants were diverse in age and gender identity, with an average length of employment being 8.5 years, ranging between 1 year and 25 years. The following three themes emerged from our findings as having a noticeable impact on staff motivation, morale, delivery of care, and client outcomes: transparent and open communication; opportunities to collaborate in decision making; and staff recognition and appreciation (see Table 1 with example quotes).

**Table 1.** Main themes with examples (*n* = 16 interviews)

Theme	Examples
Transparency and open communication (*n* = 16/16 transcripts, with 26 total mentions)	*'* […] *lot of decisions that were taken at the upper-management level, and it was not clear how those decisions were taken.'* (A0590000) *'When decisions are being contemplated, it’s usually discussed with the team, we understand the rationale, we have the opportunity to give some input, and then it does seem to roll along pretty well.'* (A0480000B) *'it helps when everybody is on the same page and everybody is agreeable to the plan … it shows respect across team members that we work together and agree on this together, and obviously it’s going to help with patient care.'* (DM200025)
Opportunities to collaborate in decision making (*n* = 16/16 transcripts, with 56 total mentions)	*'input of people who actually have to do the work day-to-day is probably the most effective thing that can be done.'* (A0560000) *'Collaboration is really key. Letting people use their voices. We don’t always have to agree, but we can — there is something that we can agree on, we can come to consensus on something.'* (A0590000) *'I feel appreciated and valued for what I contribute to my team. I have a voice. We meet often for case management, we’re consulting all the time, we’re addressing problems as a team, coming up with solutions. We make decisions together as a team, and really it increases my motivation, my commitment, my loyalty to the care that I provide and the place that I work for.'* (A0450000) *'the team is just comfortable with bringing up concerns with each other and with management when needs, because we know that they’ll be listened to.'* (A200023) *'When we are given the opportunity to provide feedback or review our concerns, then I think that you have more satisfaction.'* (A054000)
Staff recognition and appreciation (*n* = 15/16 transcripts, with 22 total mentions)	*'* […] *we have what we call Moments of Excellence, so people bring forward what they feel a moment of excellence is for a staff member, so they get acknowledged just within their own team. We also do that at staff meetings too.* […] *— as an organisation, they do a lot for staff appreciation and make them aware of how appreciated they are.'* (DM200021) *'Our manager often will take time to highlight something that we’ve done. There’s nothing huge or nothing formal, but I think that as a team, we’re good at letting each other know when we’ve done a good job.'* (DM200023)

### Transparent and open communication

Communication and transparency were key contributors to staff members’ investment and support for organisational processes and change. When their leaders were open, transparent, and offered ongoing communication about policy, workflow, and staffing changes, then morale, motivation, and commitment improved. Staff members understood that time pressure might preclude immediate communication, but expected that the leader or manager would explain the situation when time permitted. Despite the wide diversity of staff on the team and their different skillsets and perspectives, staff considered it important that no one felt excluded or disregarded. Consistent and open communication between all team members and their leaders was cited as critical for building and maintaining trust between staff and their leaders, and overall team function.

### Opportunities to collaborate in decision making

Interview participants highlighted that the ability to share feedback and provide input on general clinical issues, case management, team functioning, workflows, and so on, was an important factor in job satisfaction, morale, and organisational culture. They also highlighted that it was the responsibility of leadership to support and implement opportunities for staff to collaborate in decision making. As such, it was important for leadership to not only be open to input and feedback but also provide opportunities to do so. As a result, staff who felt listened to and were encouraged to provide feedback felt more connected and satisfied with their work. Opportunities for feedback included morning huddles; regularly scheduled meetings; informal conversations; and discussions among all staff, including managers and individuals in leadership positions.

### Staff recognition and appreciation

Interviewees commented on the importance of recognising and appreciating the work they do, as well as their knowledge and skills. They described receiving both formal and informal recognition or validation of their work such as the celebration of staff achievements as crucial. It was also shared that when staff had the autonomy to make their own decisions regarding client care, there was a perceived feeling of trust in their knowledge, skills, and abilities. This would in turn support staff confidence, and trust in leadership and encourage staff to share their input.

## Discussion

### Summary

Governments are increasingly investing and promoting team-based care. It is therefore imperative that we understand which leadership approaches and styles are the most conducive in promoting staff satisfaction, motivation, and ultimately client care within team-based care settings.

Our study found three major themes that influenced staff motivation, morale, and client care outcomes, with elements of the TL theory reflected within each. These themes were transparent and open communication, opportunities to collaborate, and staff recognition and appreciation. Our findings indicated that when leadership was open, transparent, and provided ongoing communication (inspirational motivation), staff reported improved morale, motivation, and commitment. Additionally, improvements in organisational culture, job satisfaction, morale, and client care were also reported by staff when leaders encouraged and provided opportunities for input in the decision-making process (idealised influence and intellectual stimulation). As well as when leaders recognised and appreciated their team member’s work, knowledge, and skills (individual consideration).

### Strengths and limitations

Strengths of this article include interviewing longstanding, mature teams as well as a variety of staff roles, and building on a previous team study. These interviews were carried out before the COVID-19 pandemic, and the provision of health care has become more difficult since that time. Staff shortages and pressures may make some of the themes of TL more difficult to attain in practice than they were when the interviews were conducted. This study does not address questions about the pros and cons of a TL style, or try to understand why leaders act in a more transactional or laissez-faire manner at times. It provides limited insight into how to actually implement TL on the ground. We were not able to include any individual staff characteristics when citing quotes to protect their confidentiality.

### Comparison with existing literature

Our study underlines the importance of leadership styles and approaches in which every team member is informed, heard, and appreciated. Although, the majority of current literature is focused on the impact of leadership styles within hospital settings and non-team-based primary care, there are strong commonalities between our findings and existing literature.

Among the various leadership styles identified in the literature, TL is seen as the most favoured approach when compared with transactional, task-focused, servant, and laissez-faire type approaches and has the greatest influence on the above-mentioned outcomes.^2,3,11–22^ For example, a study conducted by Musinguzi *et al^3^* examined the relationship between three different leadership styles (transformational, transactional, and laissez-faire) with motivation, job satisfaction, and teamwork of health workers, and found that TL had a statistically significant positive relationship with these outcomes, while laissez-faire was negatively associated. The study was able to suggest that the more transformational the healthcare leader was, the more motivated, satisfied, and team-spirited the team members would be, similar to our findings. A systematic review that explored different leadership styles that could be adopted by clinical staff transitioning to a leadership role found that the TL style was again more positively associated with employee retention, organisational commitment, and job satisfaction when compared with others.^11^

With regard to staff recognition and appreciation, participants in our study voiced appreciation for their leaders when they recognised their work and contributions. A handful of studies highlighted similar sentiments that were also linked to greater job satisfaction^13,23,24^ as well as lower intent to leave, staff burnout, and turnover.^1,21,22^ A review that looked at the impact of different leadership styles on burnout, absenteeism, and turnover found that TL compared with other models had the greatest impact on reducing these outcomes. The review commented that this was owing to TL stressing the importance of recognising and valuing the strengths of team members.^1^ A systematic review examined the relationship between managers’ leadership practices and nurses’ intent to stay. It found that nurses employed in the following environments were more likely to remain in their positions, and were more satisfied and more committed to the organisation:^2^ where they felt supported by their managers and peers; where they were autonomous in practice; where they felt recognised and valued for their contributions; where they were encouraged to participate in decision making; and where they were empowered to reach their full potential.

Although our study only briefly touched on the impact of leadership on client care and outcomes, a number of studies have explored this relationship.^18,23,25–27^ According to one systematic review,^1^ the attributes of TL theory are essential for promoting a culture of safety,^28^ reducing the number of adverse events^29^ as well as improving the quality of care provided to patients.^30^ A few other studies noted that using the TL style led to employee autonomy, psychological wellbeing, and inspiration all of which were cited as important in providing quality care to patients.^18,26^

### Implications for research and practice

This study provided insight on the elements of TL theory that influenced staff satisfaction, motivation, and client outcomes within a team-based model of primary health care. The findings of this study are relevant for newly forming primary healthcare teams who do not have experience with working in a team-based environment, in which leaders will have to manage a team of providers with different skillsets and perspectives. However, further research following the pandemic may provide a more accurate and current view of the importance of leadership styles in primary care. Additional research should also focus on the impact of leadership on various different roles within team-based primary care.

## Data

The dataset relied on in this article is available from the corresponding author on reasonable request.
